# Soil resource availability is much more important than soil resource heterogeneity in determining the species diversity and abundance of karst plant communities

**DOI:** 10.1002/ece3.8285

**Published:** 2021-10-28

**Authors:** Yuan Liu, Wenchao Qi, Danni He, Yunrong Xiang, Jinchun Liu, Huimin Huang, Miao Chen, Jianping Tao

**Affiliations:** ^1^ Key Laboratory of Eco‐environments in Three Gorges Reservoir Region (Ministry of Education) Chongqing Key Laboratory of Plant Ecology and Resources Research in Three Gorges Reservoir Region School of Life Sciences Southwest University Chongqing China; ^2^ State Key Laboratory of Hydraulic Engineering Simulation and Safety Tianjin University Tianjin China; ^3^ Chongqing Jinfo Mountain Karst Ecosystem National Observation and Research Station Southwest University Chongqing China

**Keywords:** community abundance, karst shrubland and woodland, soil resource availability, soil resource heterogeneity, species diversity

## Abstract

Resource availability and heterogeneity are recognized as two essential environmental aspects to determine species diversity and community abundance. However, how soil resource availability and heterogeneity determine species diversity and community abundance in highly heterogeneous and most fragile karst landscapes is largely unknown. We examined the effects of soil resource availability and heterogeneity on plant community composition and quantified their relative contribution by variation partitioning. Then, a structural equation model (SEM) was used to further disentangle the multiple direct and indirect effects of soil resource availability on plant community composition. Species diversity was significantly influenced by the soil resource availability in shrubland and woodland but not by the heterogeneity in woodland. Abundance was significantly affected by both soil resource availability and heterogeneity, whereas variation partitioning results showed that soil resource availability explained the majority of the variance in abundance, and the contribution of soil resource heterogeneity was marginal. These results indicated that soil resource availability plays a more important role in determining karst plant community composition than soil resource heterogeneity. Our SEMs further found that the multiple direct and indirect processes of soil resource availability in determining karst species diversity and abundance were different in different vegetation types. Soil resource availability and heterogeneity both played a certain role in determining karst plant community composition, while the importance of soil resource availability far exceeded soil resource heterogeneity. We propose that steering community restoration and reconstruction should be highly dependent on soil resource availability, and multiple direct and indirect pathways of soil resource availability for structuring karst plant communities need to be taken into account.

## Graphical abstract

Both limited resource availability and significant resource heterogeneity are key features of karst landscapes. Our study first considered simultaneously resource availability and heterogeneity in karst regions and then quantified and compared the effects of resource availability and heterogeneity on community abundance and species diversity from the relevance‐theoretic perspective. More importantly, our study has important guiding significance to the vegetation restoration.

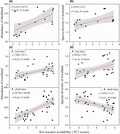

## 1 INTRODUCTION

Understanding the mechanisms that maintain vegetation is an essential ecological goal for the sustainable development of fragile ecosystem. Two general ecologically based hypotheses related to resources have been developed to explain plant community composition at the local scale. The resource heterogeneity hypothesis suggests that species diversity is a function of heterogeneity in resources because of species specialization in heterogenetic resource gradients (Huston, 1979; Ricklefs, 1977; Scott & Baer, 2019; Tews et al., 2004). Resource heterogeneity generally increases niche diversity and provides opportunities for speciation events, allowing for increasing species coexistence (Do Carmo et al., 2016; Feeser et al., 2018; Rosenzweig, 1995; Silvertown, 2004). But this is not true in small spatial patches, where there is a nonsignificant or even negative heterogeneity–diversity relationship (Gazol et al., 2013; Tamme et al., 2010; Wijesinghe et al., 2005). In contrast, the resource availability hypothesis does not necessarily rely on assumptions about species specializations and proposes that the average level of limiting resources should govern species coexistence (Bakker et al., 2003; Kumar et al., 2018; Stevens & Carson, 2002; Wassen et al., 2005; Wright, 1983). In general, under a low resource availability, the species diversity and abundance of a plant community are lower because only a few species possibly survive and grow under such harsh environments (Comita et al., 2007; Désilets & Houle, 2005).

Recent evidence has indicated that the relative importance of resource availability and heterogeneity in influencing species composition is different in different plant communities. For instance, Bartels and Chen (2010) demonstrated that resource availability drives species diversity in both young and mature stands of forest ecosystems, whereas resource heterogeneity dominates in old‐growth stands. Shirima et al. (2016) suggested that the mean soil nutrient availability explains considerable variations in tree species richness in moist forests, while vertical soil nutrient heterogeneity is a predictor of tree species richness in miombo woodlands. In fact, species diversity and abundance are scarcely the results of a single factor and direct process, with respect to either resource availability or resource heterogeneity (Whittaker et al., 2001). Soil depth varies with topographic position, which influences species diversity (Baer et al., 2005). Soil acidity has an indirect effect on plant community composition via its effects on the availability of key mineral nutrients (Chen et al., 2004; Zhu et al., 2013). Soil nitrogen increases with stand development due to increased nitrogen fixation with stand age (Hume et al., 2016). Therefore, there is a suite of direct and indirect environmental factors that simultaneously influence the species diversity and abundance of karst plant communities, but their direct and indirect relationships remain poorly understood.

Karst is an important component of global terrestrial ecosystems with the most fragile ecological environments (Ford & Williams, 2007; Huang & Cai, 2007). Fragmentation of karst landscapes is much higher than other terrestrial ecosystems, where a shallow and discontinuous soil layer with exposed rocks forms various habitats, such as rocky fissures, rocky gullies, soil faces, rocky faces, and rocky pores (Chen et al., 2016; Nie et al., 2011; Zhang et al., 2007). The broken terrain dramatically increases the spatial variability of other environmental variables, notably the physical and chemical properties of the soil (Toure et al., 2015; Zhong et al., 2014). Additionally, previous researches have demonstrated that the spatial distribution of soil resource varied obviously in karst region (Tateno & Takeda, 2003; Zhang et al., 2006). Therefore, the high edaphic heterogeneity provides numerous ecological niches for plant diversification and speciation (Wang et al., 2014); species diversity and abundance of karst plant community are expected to be determined by soil resource heterogeneity. Nevertheless, the soil resource shortage also is a remarkable feature of karst landscapes owing to slow soil formation, a shallow soil layer and severe soil erosion, so the species diversity and abundance of karst plant community are likely to be strongly influenced by the availability of one or more limiting soil resource. As consequence, karst landscapes not only represent limited soil resource availability but also exhibit significant soil resource heterogeneity, the effect of soil resource availability and heterogeneity on karst species diversity must be taken into consideration simultaneously. In prior studies of karst ecosystem, the fact that some soil resource availability (e.g., soil depth, P availability, K availability) limits species diversity and abundance has been widely demonstrated (Crowther, 1982; Liu et al., 2020; Toure et al., 2015; Zhang et al., 2013). However, few studies have examined the effects of small‐scale soil resource heterogeneity on karst plant community species composition; and no study has examined the role of soil resource availability and heterogeneity in karst plant species diversity and abundance.

Here, we chose six sites typical in karst environment in southwestern China to quantify and compare the effects of soil resource availability and heterogeneity on species diversity and abundance. We then further disentangled the direct and indirect effects of soil resource availability on karst plant community composition by SEM. We addressed the following questions: (1) How do soil resource availability and heterogeneity affect both species diversity and abundance in the highly heterogeneous and most fragile karst mountains? (2) What is the relative importance of soil resource availability and heterogeneity for shaping karst plant community species composition? (3) How is karst plant community species composition driven by multiple direct and indirect pathways of soil resource availability?

## 2 MATERIALS AND METHODS

### 2.1 Study site

**FIGURE 1:**
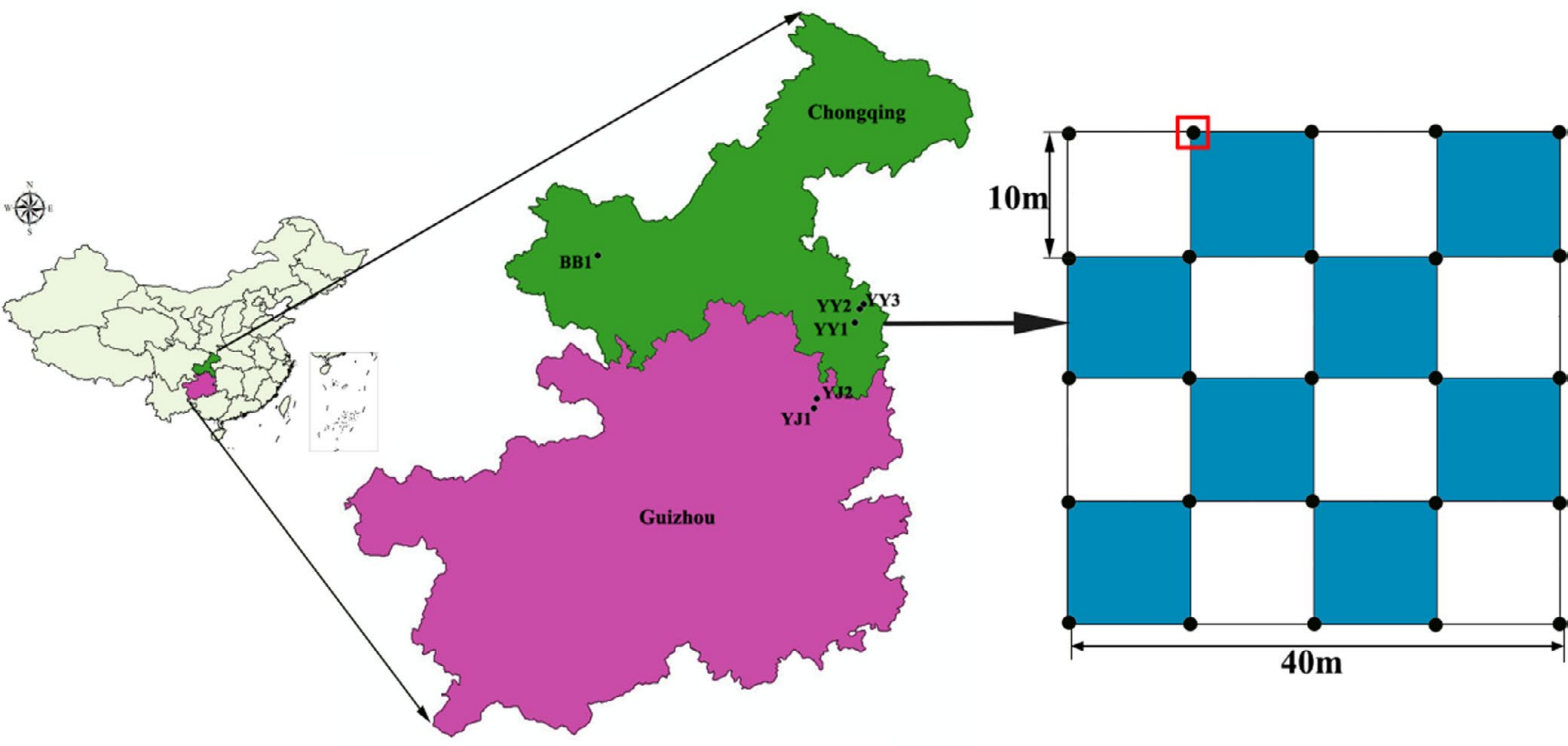
Location and layout of sample plot in the three typical karst districts of southwest China. BB1 is in Beibei; YJ1 and YJ2 are in Yinjiang; and YY1, YY2, and YY3, and YY3 are in Youyang. YY1 and YY2 belong to shrubland; BB1, YJ1, YJ2, and YY3 belong to woodland. Blue gird showed investigated units of vegetation, and red bounding box represents 2 × 2 m quadrats for measuring variables

This study was conducted from July 2017 to October 2017 at six study sites, which extend from 28°4′ to 29°36′N latitude and from 106°28′ to 108°57′E longitude and are located in three typical karst districts of southwestern China (Youyang (YY) in southeastern Chongqing; Yinjiang (YJ) in northern Guizhou Province; and Beibei (BB) in northwestern Chongqing) (Figure 1 and Table 1). The type of soil in the three districts is limestone soil, which was derived from Triassic limestone. The three districts are separated by approximately 100~250 km and are characterized by a typical subtropical monsoon climate, with mean annual precipitation and evaporation of 1200 and 1177 mm, respectively, and the average temperature ranges from 6.4°C in the coldest month (January) to 29.1°C in the warmest month (July), with a mean annual temperature of 18.5°C.

**TABLE 1:** General description of study sites

Study sites	Latitude /°N	Longitude /°E	Altitude /m	Aspect	Slope/°	Forest types	Soil type	Location
YY1	29°00´33″	108°58´5″	475	EN	23°	Shrub	Limestone	Youyang
YY2	29°00´46″	108°57´29″	479	ES	22°	Shrub	Limestone	Youyang
YY3	28°58´40″	108°57´4″	765	SW	24°	Forest	Limestone	Youyang
YJ1	28°4´6″	108°31´46″	833	NW	23°	Forest	Limestone	Yinjiang
YJ2	28°4´16″	108°32´19″	891	NW	22°	Forest	Limestone	Yinjiang
BB1	29°16´4″	106°28´2″	688	NW	20°	Forest	Limestone	Beibei

The six study sites, which include two natural secondary shrublands (YY1 and YY2) and four natural secondary woodlands (BB1, YJ1, YJ2, and YY3), were selected in the study area. The forests in these sites are strictly protected against disturbance by the ways of closing and setting apart hills for plants growing, especially the Chinese government began to perform the National Natural Forest Protection Project in these areas after 1998. The elevation of different shrublands and woodlands ranges between 476–479 m asl and 683–851 m asl, respectively. Besides, different shrublands or woodlands are located on gentle slope of typical karst mountains where the slope, aspect, and inclination are basically the same (Table 1). The shrubland is dominated by drought‐tolerant plants, and the most common species include *Berchemia sinica* Schneid., *Rhamnus leptophylla* Schneid., *Rhus chinensis* Mill., *Rosa cymosa* Tratt., and *Pyracantha fortuneana* (Maxim.) Li. The dominant tree species in woodland areas are *Pinus massoniana* L., *Cupressus funebris* Endl., and *Cunninghamia lanceolata* (Lamb.) Hook. Common understory shrubs in woodlands are *Myrsine africana* Linn., *Viburnum chinshanense* Graebn., *Smilax china* L., *Lindera glauca* (Sieb. et Zucc.) Bl., and *Rosa cymosa* Tratt. In total, 116 species were found in the study (a list of the most common species and their frequency can be found in Table S1). For nomenclature of the species recorded, see the Flora of China (Wu et al., 2013).

### 2.2 Field data collection

We established a 40 m × 40 m sample plot at each study site. Each sample plot was divided into 16 equal‐sized grid cells of 10 m × 10 m (Figure 1). The interval's grid cell of 10 m × 10 m were selected as the vegetation quadrats, in which all trees and shrubs were investigated (Blue gird cell in Figure 1). Therefore, each 40 m × 40 m sample plot contains eight 10 m × 10 m vegetation quadrats and total 32 vegetation quadrats in woodlands and 16 vegetation quadrats in shrublands were investigated. We then set 2 × 2 m soil quadrats at four corners of the vegetation quadrats, from which soil depth (SD), rock bare rate (proportion of exposed rock) (RBR), and elevation were recorded. The rock bare rate was estimated visually by three observers, elevation was measured using a hand‐held GPS, and soil depth was measured by vertically inserting a single‐headed, sharp steel rod at the center and four corners of each 2 × 2 m quadrat until the rock was reached.

### 2.3 Soil sampling and laboratory analyses

Soil moisture (W) was quantified by an oven‐dry method, and soil bulk density (ρb) was quantified by a cutting ring (5‐cm diameter and 5‐cm high) in each 2 × 2 m quadrat. Five soil samples were collected at a depth of approximately 15 cm from the center and four corners of each 2 × 2 m quadrat and mixed into a single composite sample. The composite soil samples were air‐dried at room temperature, ground, and passed through a 100‐mesh plastic sieve to determine the chemical properties.

Soil chemical properties including pH, total carbon (TC), total nitrogen (TN), total phosphorus (TP), total potassium (TK), available nitrogen (AN), available phosphorus (AP), available potassium (AK), calcium (Ca), and magnesium (Mg) were analyzed for each quadrat using standard soil test methods (Liu et al., 2012). Soil pH was measured in a 1:2.5 (w/w) ratio of soil and deionized water at 20°C. Soil TC and TN concentrations were determined by combustion on a Vario EL cube CN Elemental Analyzer (Elementar Analysen Systeme GmbH). After acid digestion in a mixture of HNO_3_, H_2_O_2,_ and HF (7:2:1) in a microwave digestion system, soil TK, Ca, and Mg concentrations were determined by an inductively coupled plasma mass spectrometer (ICP Spectrometer, Thermo Scientific), and soil TP was measured by an ultraviolet spectrophotometer (UV2550, Shimadzu). Soil AN was quantified using the diffusion–absorption method. Soil AK was measured as described for TK measurement after extraction using 1.0 mol/L CH_3_COONH_4_ solution. Soil AP was determined via molybdenum–antimony colorimetry after extraction using 0.5 mol/L NaHCO_3_ solution (UV2550 Spectrophotometer, Shimadzu).

### 2.4 Statistical analyses

The abundance of the plant community was determined by the total number of individuals in each 10 × 10 m grid cell. The species diversity of each 10 × 10 m grid cell was calculated using the Shannon–Weiner index ($H=‐\sum_{i=1}^{S}\frac{N_{i}}{N}\log_{2}\frac{N_{i}}{N}$, where *N_i_* is the number of individuals of the *i*th species, and *N* is the total number of individuals of all species) (Magurran, 2004). The availability and heterogeneity of each variable for the 10 × 10 m grid cell were calculated using four measurements from the 2 × 2 m quadrat of each grid cell, the mean of four measurements was used to express soil resource availability, and the coefficient of variation (CV) was employed as the measure of soil resource heterogeneity (Marchand & Houle, 2006; Reynolds et al., 2007; Shirima et al., 2016; Ulrich et al., 2018).

Prior to further statistical analysis, all data were checked for normality using the Shapiro–Wilk test and homogeneity of variance using Levene's test. Any data that did not meet normality and homogeneity of variance were transformed using an appropriate method. All above‐mentioned analyses were performed using SPSS 13.0 (SPSS Inc. IL, USA). The influence of elevation on the plant community can be neglected in this study because the elevation of different plant communities has a small change with 17 m in shrubland and 177 m in woodland (Table 2). Therefore, in the following data analyses, elevation was not included among the variables.

**TABLE 2:** Summary of the measured environment variables, mean (arithmetic mean for 10 × 10 m grid cell), range (minimum and maximum values for 10 × 10 m grid cell), and mean CV (arithmetic mean of variation coefficient for 10 × 10 m grid cell) in karst shrubland and woodland

Variables	Shrubland			Woodland		
	Mean	Range	Mean CV	Mean	Range	Mean CV
Elevation (ELE, m)	478	470~487	0.004	762	680~857	0.002
Soil depth (SD, cm)	10.616	3.437~18.860	0.384	18.704	7.964~39.259	0.247
Rock bare ratio (RBR, %)	53.135	21.417~80.917	0.437	32.292	0.417~68.250	0.625
pH	6.420	6.038~6.678	0.032	5.738	5.110~6.723	0.048
Total carbon (TC, g/kg)	31.509	23.650~42.950	0.208	37.275	20.200~61.825	0.199
Total nitrogen (TN, g/kg)	4.644	2.500~6.900	0.330	3.191	1.825~5.350	0.172
Total phosphorus (TP, g/kg)	0.349	0.221~0.473	0.287	0.212	0.094~0.392	0.205
Total potassium (TK, g/kg)	19.913	14.338~25.805	0.097	14.868	10.818~18.163	0.063
Available nitrogen (AN, mg/kg)	198.037	105.112~288.512	0.329	221.359	89.712~485.212	0.263
Available phosphorus (AP, mg/kg)	3.268	2.001~4.320	0.530	4.051	0.853~16.709	0.768
Available potassium (AK, mg/kg)	124.882	89.450~166.525	0.213	107.887	71.150~179.025	0.210
Calcium (Ca, g/kg)	4.480	3.453~5.833	0.242	4.294	1.207~12.000	0.261
Magnesium (Mg, g/kg)	5.702	5.147~7.062	0.109	5.122	2.644~8.760	0.094
Water content (W, %)	27.135	14.823~36.765	0.126	30.634	24.168~38.745	0.140
Bulk density (ρb, g/cm^3^)	1.496	1.319~1.668	0.062	1.513	1.324~1.683	0.065

To test the effects of soil resource availability and heterogeneity on species diversity and abundance in different vegetation types, we first quantified the main axes of availability and heterogeneity of overall variables using principal component analysis (PCA). The first axis scores of the PCA were used as a multivariate proxy for availability and heterogeneity of overall variables. Then, we tested the relationship between species diversity and abundance with the first PCA axis scores of soil resource availability and heterogeneity by linear regression. The PCA was performed using the “princomp” function in the R stats package (R Core Team, 2014). Linear regression and Pearson's correlation analysis were performed using Origin 8.5 (Origin, Northampton, MA, USA).

The variation partitioning approach was used to analysis the relative influence of soil resource availability and heterogeneity on community species composition. The variation of community species composition was partitioned into the components explained by soil resource availability and heterogeneity together, as well as the components explained by each of these independently. Before variation partitioning, forward selection was applied within each of the two sets of explanatory variables to identify the significant environmental factors (Blanchet et al., 2008). The variation in species composition was decomposed in R 3.5.3 using the “varpart” function of the vegan package (Oksanen et al., 2018), and forward selection was performed in R 3.5.3 using the “forward.sel” function of the adespatial package (Dray et al., 2017).

Finally, we used a structural equation model (SEM) to explore the direct and indirect relationships between the soil resource availability and species diversity and abundance for different vegetation types. SEM was used because it enables the testing of direct and indirect hypothesized relationships among the variables and provides more insights into complex systems than univariate analyses (Kubota et al., 2004). Initially, all plausible interaction paths among all variables were considered in a full model. Then, some modified models were developed by removing direct and indirect pathways with low and nonsignificant path coefficients until an adequate fit was obtained (Grace et al., 2010). All direct and indirect path coefficients (λ) were standardized regression coefficients, and the pathways of SEMs were significant if *p < .05* of the standardized regression coefficient (Désilets & Houle, 2005; Kumar et al., 2018; Sande et al., 2017). The relative importance of causal factors for species diversity and abundance was compared using the total effect from direct and indirect effects. The goodness of fit for the working model was determined by the maximum‐likelihood chi‐squared statistic (χ^2^), the comparative fit index (CFI), and the standardized root‐mean‐square residual (SRMR). The model was judged as a reasonable fit if *p* > .05, which indicates that fitting covariance matrices are not significantly different from observed covariance matrices (Grace et al., 2010). CFI > 0.95 suggests a very good fit, which is little affected by sample size compared to the chi‐square test (Rosseel, 2012). SRMR ≤ 0.05 indicates a very close fit between a model and the observed data (Browne & Cudeck, 1992). All models were implemented in R 3.5.3 using the “sem” function of the lavaan package (Rosseel et al., 2020).

## 3 RESULTS

### 3.1 Effects of soil resource availability and heterogeneity on species diversity and abundance

The first PCA axis (PC1) accounted for 40% of the soil resource availability variation for shrubland, 55% of that for woodland, and 30% of the soil resource heterogeneity variation for both shrubland and woodland; moreover, the PC1 eigenvectors of both soil resource availability and heterogeneity were greater than 2.00; therefore, PC1 explained the majority of the variability in the soil resource availability and heterogeneity in this study (Figure S1, Table S2).

**FIGURE 2:**
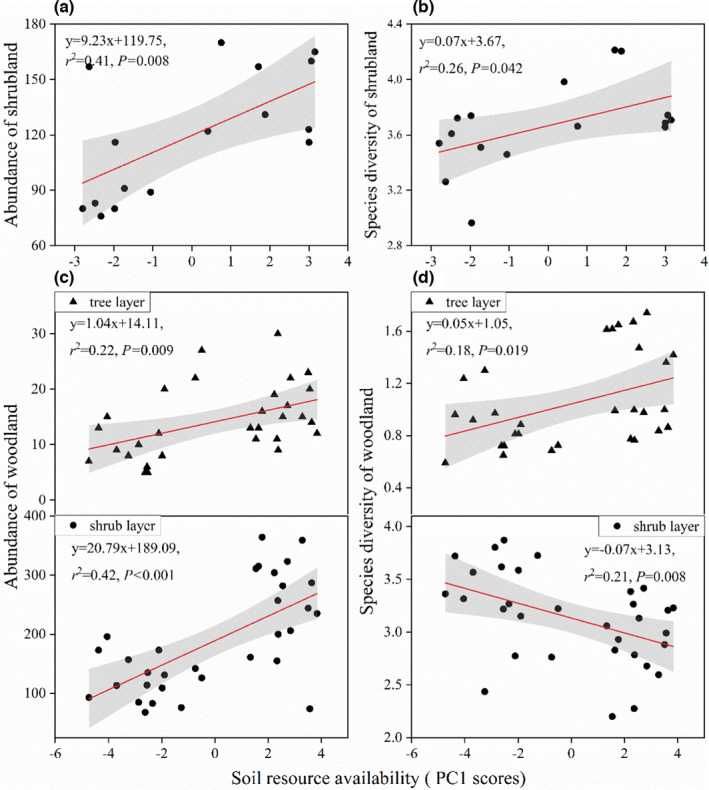
Relationships of soil resource availability PC1 scores with (a, c) plant abundance in karst shrubland and woodland, and (b, d) species diversity in karst shrubland and woodland. (c) and (d) included tree and shrub layers of woodland, respectively. Statistical significance of the regression models indicated by * at *p* < .05 and ** at *p* < .01, and gray shading represents 95% credible intervals

**FIGURE 3:**
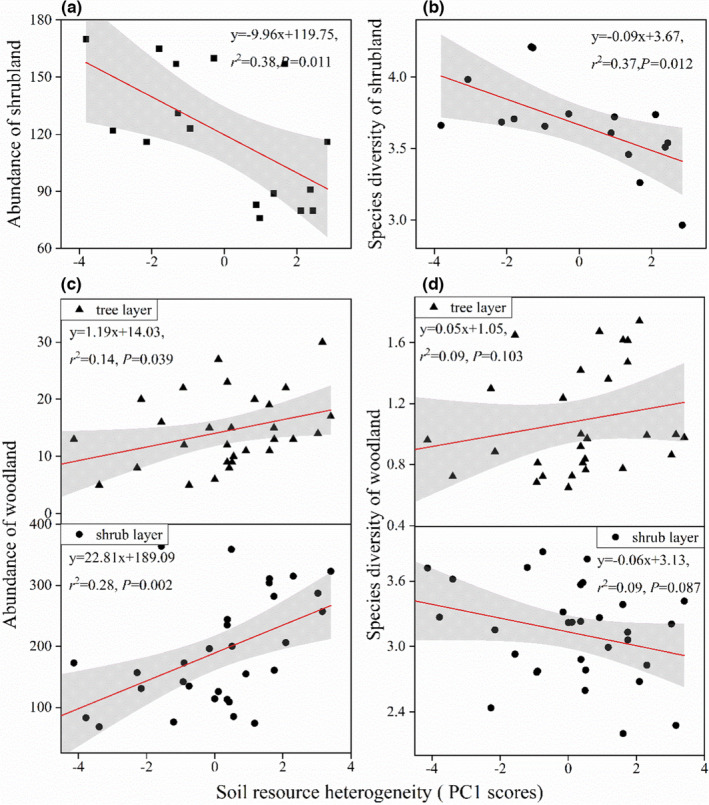
Relationships of soil resource heterogeneity PC1 scores with (a, c) plant abundance in karst shrubland and woodland, and (b, d) species diversity in karst shrubland and woodland. (c) and (d) included tree and shrub layers of woodland, respectively. Statistical significance of the regression models indicated by * at *p* < .05 and ** at *p* < .01, and gray shading represents 95% credible intervals

With increasing soil resource availability, species diversity and abundance significantly increased in both shrubland and woodland except species diversity in the shrub layer of woodland (Figure 2a–d). Species diversity and abundance were both significantly negatively related to soil resource heterogeneity in shrubland (Figure 3a,b). However, community abundance significantly increased with increasing soil resource heterogeneity in woodland (Figure 3c). There was no significant relationship between species diversity and soil resource heterogeneity in woodland (Figure 3d).

### 3.2 The relative importance of soil resource availability and heterogeneity in plant abundance

**FIGURE 4:**
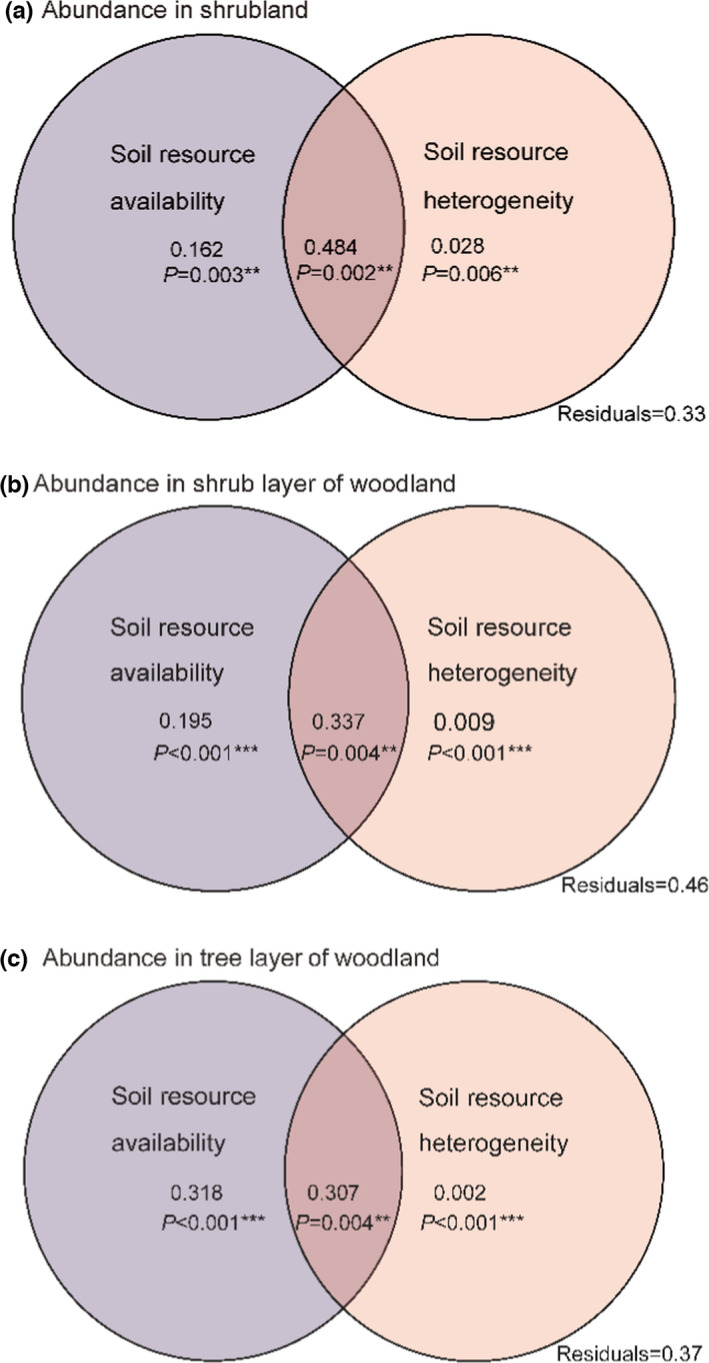
Variation partitioning of (a, b, c) abundance explained by soil resource availability and heterogeneity in three vegetation types, (a) for shrubland, (b) for tree layer of woodland, and (c) for shrub layer of woodland. The coefficients of explained variation (R^2^) are very significant (*p* < .01)

The total variation in community abundance explained by both soil resource availability and heterogeneity was 67% for shrubland and 64% and 63% for shrub layer and tree layer in woodland (Figure 4). The shared fraction between soil resource availability and heterogeneity explained a large proportion of the variation in community abundance (48.4%, *p* = .002 in shrubland; 33.7%, *p* = .004 and 30.7%, *p* = .004 in the respective tree layer and shrub layer in woodland). Abundance was also largely explained by the unique contribution of soil resource availability (16.2%, *p* = .003 in shrubland; 19.5%, *p* < .001 and 31.8%, *p* < .001 in the respective tree layer and shrub layer in woodland), but the unique contribution of soil resource heterogeneity was marginal (2.8%, *p* = .006 in shrubland; 0.9%, *p* < .001 and 0.2%, *p* < .001 in the respective tree layer and shrub layer in woodland) (Figure 4).

### 3.3 Multiple direct and indirect effects of soil resource availability on species diversity and abundance

**FIGURE 5:**
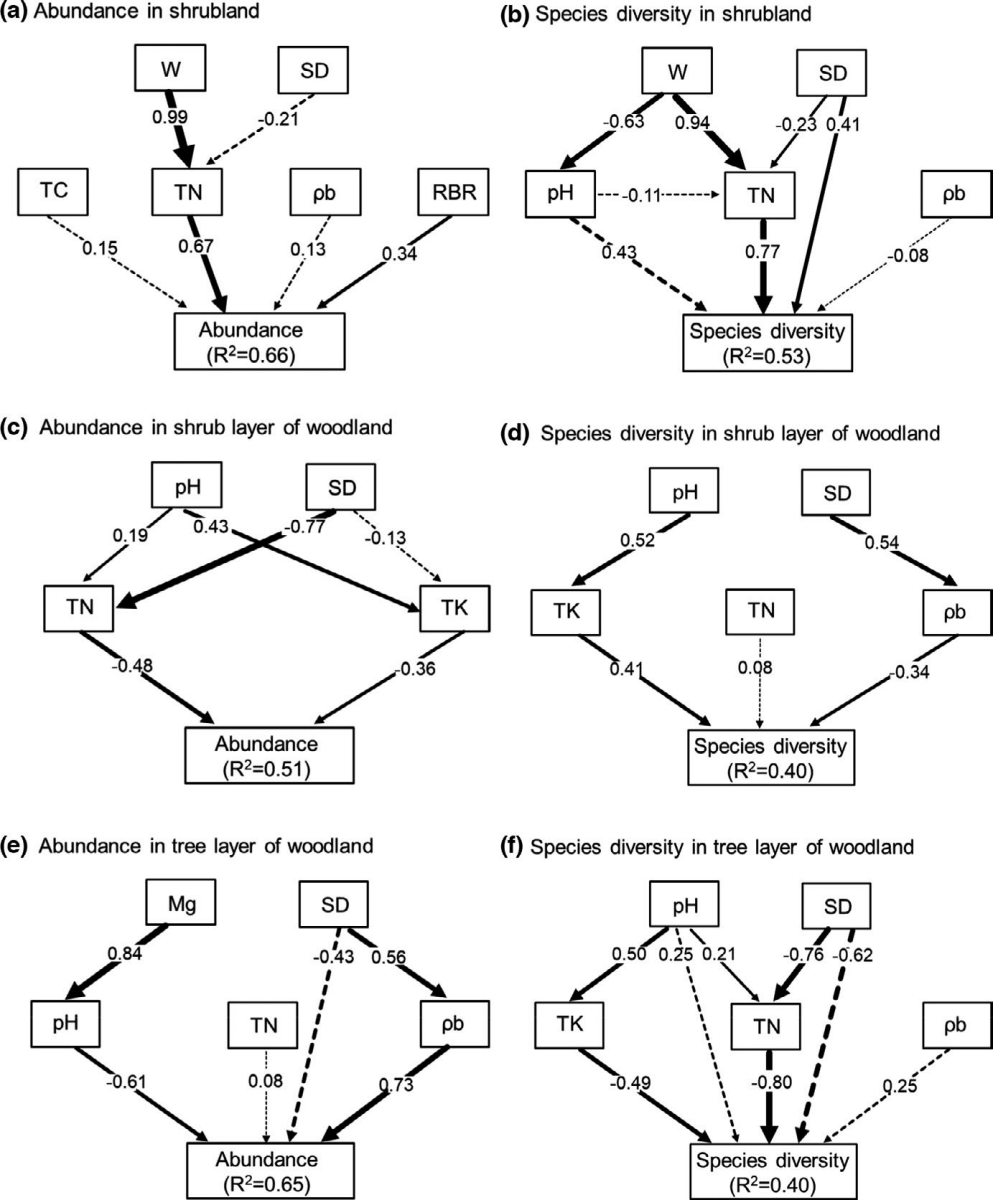
Structural equation model (SEM) linking each soil resource availability to plant (a) abundance and (b) species diversity in shrubland, (c) abundance and (d) species diversity in shrub layer of woodland, and (e) abundance and (f) species diversity in tree layer of woodland. The numbers above the arrows indicate path coefficients (λ, standardized regression coefficients), and the strength of path coefficients is indicated by the width of the arrows. Solid black lines indicate significant (*p* < .05) pathways, and dashed black lines indicate nonsignificant (*p* > .05). *R* ^2^ values represent the proportion of variance explained for abundance and species diversity. For abbreviations, see Material and Methods and Table 2

To further disentangle the complex relationship between soil resource availability and plant community characteristics, SEMs were carried out for each vegetation type. Our SEMs (Figure 5a–f) produced good fits with our data for community abundance and species diversity in shrubland and woodland (Table 3). Therefore, these models successfully assessed the extent to which factors influenced species diversity and abundance through direct and indirect pathways.

**TABLE 3:** The goodness of fit for the working SEMs

Vegetation types	Abundance					Species diversity				
	χ^2^	*df*	*p*	CFI	SRMR	χ^2^	*df*	*p*	CFI	SRMR
Shrubland	1.229	4	.873	1.000	0.019	3.873	4	.424	1.000	0.060
Shrub layer of Woodland	2.757	3	.431	1.000	0.029	8.257	7	.310	0.962	0.050
Tree layer of Woodland	4.341	6	.631	1.000	0.062	3.418	4	.490	1.000	0.047

Abbreviation: CFI, the comparative fit index; SRMR, the standardized root‐mean‐square residual; χ^2^, the maximum‐likelihood chi‐squared statistic.

**FIGURE 6:**
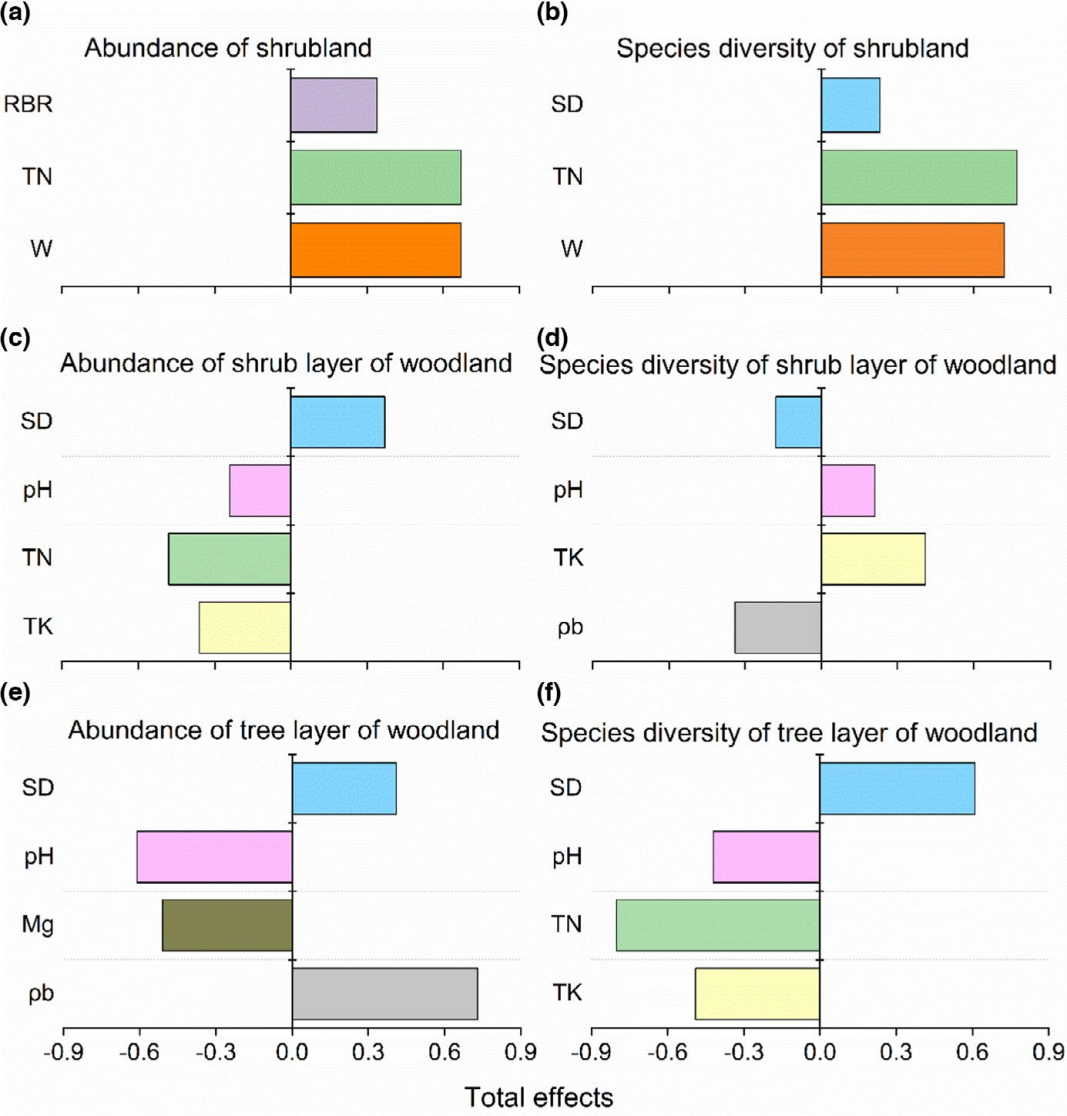
Coefficients of standardized total effect (direct plus indirect effect) of each variable on plant (a) abundance and (b) species diversity in shrubland, (c) abundance and (d) species diversity in shrub layer of woodland, (e) abundance and (f) species diversity in tree layer of woodland derived from structural equation model (SEMs). All variables in the above figures are significant (*p* < .05). For abbreviations, see Material and Methods and Table 2

In shrubland, the standardized effect of environmental factors derived from SEMs showed that soil TN had positive direct effects on both shrub abundance (standardized coefficient, λ = 0.67) and species diversity (λ = 0.77). Soil moisture had positive indirect effects on both shrub abundance (λ = 0.67) and species diversity (λ = 0.72) via soil TN. Therefore, the soil TN and soil moisture are the strongest predictor of shrub abundance and species diversity. Additionally, soil depth had a significantly positive effect on species diversity by direct and indirect effects (Figures 5a,b and 6a,b, Table S3).

For the shrub layer in woodland, soil depth had positive indirect effect (λ = 0.37), and soil pH had negative indirect effects (λ = −0.24) on plant community abundance through TN and TK. Additionally, soil nutrient factors all had negative direct effects (TN: λ = −0.48; TK: λ = −0.36) on abundance (Figures 5c and 6c, Table S4). In contrast, soil depth had negative indirect effect (λ = −0.18), and soil pH had positive indirect effects (λ = 0.21) on species diversity. Species diversity was mainly positively explained by soil TK (λ = 0.41) (Figures 5d and 6d, Table S4).

For the tree layer in woodland, plant community abundance was mainly influenced by soil ρb (λ = 0.73), followed by soil pH (λ = −0.61), Mg (λ = −0.51), and depth (λ = 0.41), among which the negative effect of soil Mg on abundance was through indirectly effecting soil pH. The direct effect of soil depth on abundance was nonsignificant, but soil depth had positive indirect effect on abundance via ρb (Figures 5e and 6e, Table S4). Overall species diversity was primarily driven by soil TN (λ = −0.80), depth (λ = 0.61), TK (λ = −0.49), and pH (λ = −0.42). Soil depth and soil pH indirectly influenced species diversity via soil nutrient factors, and soil TN and TK had direct negative effect on species diversity (Figures 5f and 6f, Table S4).

## 4 DISCUSSION

### 4.1 Soil resource availability plays a large role for shaping plant community species composition

Our findings highlight that species diversity and abundance were both significantly associated with soil resource availability in karst ecosystem (Figure 2a–d), which completely supports the resource availability hypothesis. Similar results have proven that the availability of single variables (soil organic matter, phosphorus, nitrogen, etc.) was closely related to species diversity (Ou et al., 2014), but our study further focused on the effect of integrated environmental variables on species diversity. It is important to note that the total amount of soil resources in karst regions is very limited because of the slow and patchy development of nutrient‐poor soil over exposed rock (Kavouri et al., 2011). Given these soil resource limitations, species diversity and abundance significantly increased with increasing soil resource availability in shrubland and the tree layer of woodland. Broadly speaking, karst woodland with better soil environmental conditions could provide enough nutrients in terms of shrub growth (Asensio et al., 2013); therefore, the negative soil resource availability–species diversity relationship was most likely because dense overstory canopy intercept available light (Neufeld & Young, 2014; Zhang et al., 2017).

Meanwhile, plant community abundance significantly increased with increasing soil resource heterogeneity in the shrub layer and tree layer of karst woodland, corroborating the findings in other ecosystems (Silvertown, 2004; Stein et al., 2014; Tilman, 1982). This is because karst woodland with low fragmentation degree have a relatively mild soil environment for plant survival and growth. Increasing heterogeneity provides enough nutrients and more niches to seed germination and plant growth, allowing for increased community abundance in woodland. Therefore, the significant relationship between soil resource heterogeneity and community abundance also favors the resource heterogeneity hypothesis. Given that plant community abundance in karst region was significantly associated with soil resource availability, as well as soil resource heterogeneity, which suggested that soil resource availability and heterogeneity as two important drivers of community abundance are not mutually exclusive in karst shrubland and woodland. Our variation partitioning further elucidate the relative importance of soil resource availability and heterogeneity in determining community abundance. It is clear that a large proportion of variation in community abundance was explained by unique soil resource availability, with only a small amount explained by unique soil resource heterogeneity (Figure 4), demonstrating that soil resource availability had a much higher explanatory power for the variation in community abundance compared with soil resource heterogeneity.

Species diversities of tree and shrub layer of woodland were not related with soil resource heterogeneity fully confirmed that soil resource availability was more significant predictor of species diversity rather than soil resource heterogeneity. This finding also corresponds with those from other ecosystems, where species diversity appeared to be determined by soil resource availability rather than soil resource heterogeneity (Baer et al., 2004; Lundholm, 2010). The degree of rock fragmentation in karst shrubland is far higher than that in karst woodland (Liu et al., 2019); thereby, plants are likely to face soil resource deficiency in the highly heterogeneous karst shrubland. Additionally, plant individuals cannot survive when the soil patch size is relatively smaller than the plant root size (Gazol et al., 2013; Lundholm, 2009; Schuler et al., 2017; Tamme et al., 2016). To the extent that the size of the root system of individual plants is more likely to exceed the soil patch size with increasing environmental heterogeneity in karst shrubland so that plants located in unfavorable soil patches face an increased risk of mortality (Laanisto et al., 2013). As a consequence, species diversity and abundance significantly decreased with increasing soil resource heterogeneity mainly because soil resource availability limits survival and growth of plant individuals in the highly heterogeneous karst shrubland, while increasing soil resource heterogeneity does not work when soil and space resource is extreme shortage in karst shrubland.

### 4.2 Multiple direct and indirect processes of soil resource availability driving plant community species composition

Our SEMs further disentangled the multiple direct and indirect processes by which species diversity was influenced by the availability of each variable. Species diversity and abundance were mainly controlled by soil TN and soil moisture in shrubland (Figures 5a,b and 6a,b, Table S3). Similar results have been found in karst region, where plant community structure was strongly influenced by soil TN during the early successional stages (Li et al., 2017; Zhang et al., 2015). In fragmented karst shrubland, a large amount of exposed bedrock and an extremely shallow soil depth lead to a shortage of soil resources; therefore, soil is highly TN deficient, which significantly influences species diversity and abundance. Additionally, the soil TN is mainly originated from the slow release of soil organic matter (Jobbágy & Jackson, 2000). However, the severe water loss along stone crevices and lots of water evaporation result in temporary droughts in karst soil patches, especially in shallow soil patches (Wang et al., 2003), which greatly affects the release processes of soil organic matter, further exacerbates the soil nutrient deficiency (Moyano et al., 2013). Thus, soil moisture had positive indirect effects on both shrub abundance and species diversity through affecting soil TN (Figures 5a,b and 6a,b, Table S3). Furthermore, soil depth had a significant positive effect on species diversity due mostly to directly affecting plant survival space and soil resource level.

As a whole, the soil nutrient level of woodlands higher than that of shrublands because a large number of litters were accumulated in karst woodland (Islam et al., 2015; Zhang & Pan, 2012); therefore, woodland could provide enough nutrients in terms of shrub survival and growth, and soil nutrient level of woodlands did not limit the increase of shrub abundance. Soil depth was the significant predictor of shrub abundance in woodland, which was attributed to the fact that shallow soil always exposes a large amount of bedrock surface, but deeper soil provides more growing space for shrubs (Lundholm, 2010). In contrast, species diversity was mainly positively influenced by soil TK and negatively influenced by soil depth (Figures 5d and 6d, Table S4), which demonstrated that plant species tend to occur and grow in shallow soils with high nutrient content. This pattern of shrub diversity in woodland was probably because other biological factors dominate the spatial distribution of different species, as well as was supported by the fact that shallow limestone soil is rich in organic matter, nutrients (N, P, and K), and Ca (Zhang et al., 2010).

For the tree layer of woodland, soil depth and pH had significant positive and negative effect, respectively, on abundance and species diversity, which indicated that the deep soil with a high nutrient level and growing space, as well as low acidity, was favorable for the growth and survival of high‐biomass wood species. Additionally, soil Mg had a significant negative influence on abundance, while the negative effect originated mostly from the indirect affecting soil pH. The result further suggested that soil pH is an important determinant for shaping tree abundance in the forest dominated by *Pinus massoniana* L., possibly because *P*. *massoniana* prefers slightly acidic soil rather than alkaline limestone soil. However, the species diversity was mainly negatively influenced by soil nutrients (TN and TK) was because of the cause that the significant negative relationship between soil nutrient content and soil depth in karst woodlands.

## 5 CONCLUSIONS

Our study provided novel insights to quantify and compare the effects of soil resource availability and heterogeneity on plant community characteristics in the most fragile karst landscapes in southwestern China. Soil resource availability and heterogeneity both played a certain role in determining karst plant community composition, while the importance of soil resource availability far exceeded soil resource heterogeneity, thereby tending to support the resource availability hypothesis. Thus, the soil resource availability of shrubland and woodland should be more important for protecting and restoring objects than soil resource heterogeneity, especially in highly fragmented shrubland. Our SEMs further demonstrated that the multiple direct and indirect processes of soil resource availability determined karst species diversity and abundance simultaneously, whereas the multiple pathways were different in different vegetation types, emphasizing that steering community restoration and reconstruction also have to take into account multiple pathways of soil resource availability for structuring different karst community types.

## CONFLICT OF INTEREST

The authors declare that they have no conflict of interest.

## AUTHOR CONTRIBUTIONS

**Yuan Liu:** Conceptualization (lead); Formal analysis (lead); Investigation (lead); Writing‐original draft (lead); Writing‐review & editing (lead). **Wenchao Qi:** Formal analysis (supporting); Visualization (equal). **Danni He:** Data curation (equal); Investigation (equal). **Yunrong Xiang:** Data curation (equal); Investigation (equal). **Jinchun Liu:** Conceptualization (equal). **Huimin Huang:** Investigation (equal). **Miao Chen:** Investigation (equal). **Jianping Tao:** Funding acquisition (lead); Project administration (lead); Supervision (lead); Validation (lead); Writing‐review & editing (lead).

## Supporting information

Appendix S1

## Data Availability

Underlying data are available on the FigShare digital repository (https://doi.org/10.6084/m9.figshare.16782463).
