# Supplementary material for: Soil resource availability is much more important than soil resource heterogeneity in determining the species diversity and abundance of karst plant communities
Source: Ecol Evol. 2021 Oct 28;11(23):16680–92. doi: 10.1002/ece3.8285 (PMC8668789; doi:10.1002/ece3.8285)
Supplement: Supplementary file 1 — Appendix S1 [file ECE3-11-16680-s001.docx]

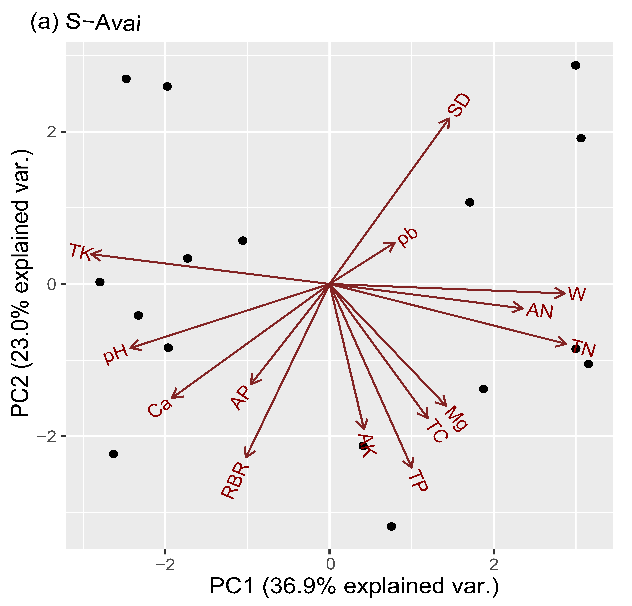

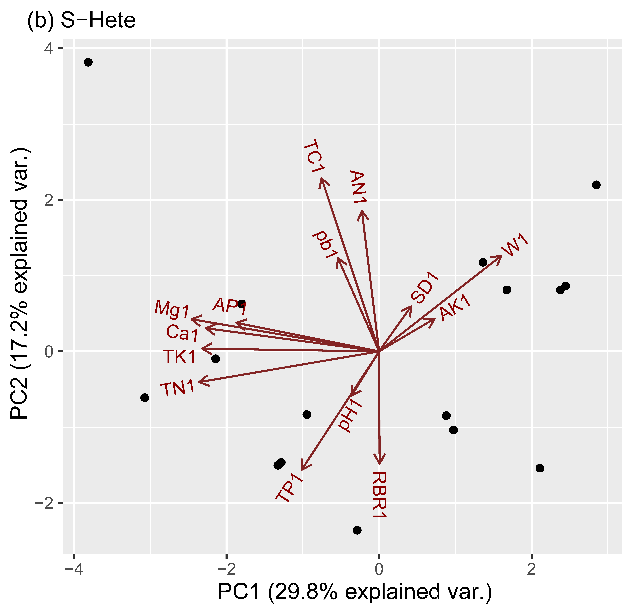


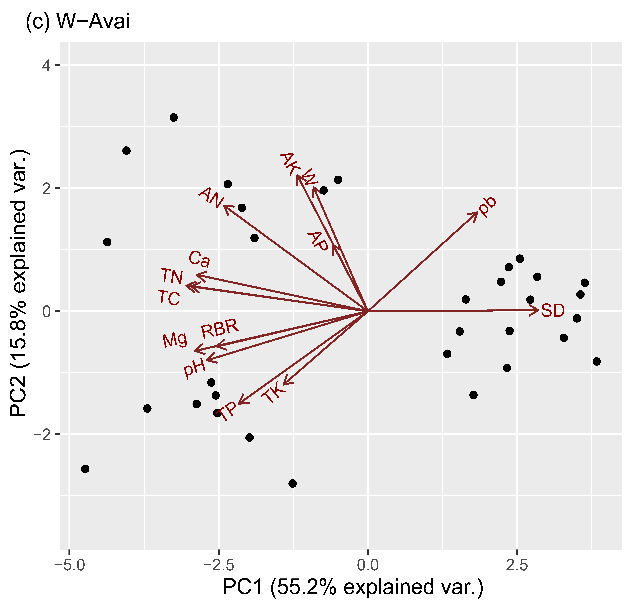

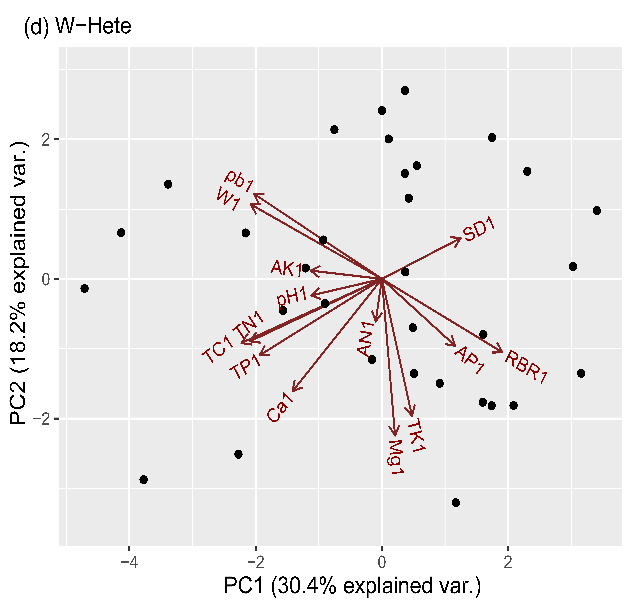


**Fig. S1** Principal Component Analysis (PCA) ordination of (a) the availability and (b) heterogeneity of each variation for karst shrubland, (c) the availability and (b) heterogeneity of each variation for karst woodland. S- Avai: soil resource availability in shrubland; S-Hete: soil resource heterogeneity in shrubland; W- Avai: soil resource availability in woodland; W-Hete: soil resource heterogeneity in woodland. Red arrows represent availability and heterogeneity of each variable for 10 × 10 m grid cell (Abbreviations, full names were in Material and Methods and Table 2).

**Table S1** The most common species occurring in the 16 shrubland samples (10 m × 10 m grid cells) and 32 woodland samples (10 m × 10 m grid cells).

| Habitat | Species | Family | Frequency |
| --- | --- | --- | --- |
| Shrubland | *Berchemia sinica* | Rhamnaceae | 1 |
|  | *Rhamnus leptophylla* | Rhamnaceae | 1 |
|  | *Rhus chinensis* | Anacardiaceae | 0.94 |
|  | *Rosa cymosa* | Rosaceae | 0.94 |
|  | *Pyracantha fortuneana* | Rosaceae | 0.94 |
|  | *Rosa roxburghii* | Rosaceae | 0.88 |
|  | *Mallotus repandus* | Euphorbiaceae | 0.88 |
|  | *Smilax china* | Liliaceae | 0.75 |
|  | *Rubus coreanus* | Rosaceae | 0.75 |
|  | *Rosa multiflora* | Rosaceae | 0.75 |
| Woodland | *Pinus massoniana* | Pinaceae | 0.94 |
|  | *Cupressus funebris* | Cupressaceae | 0.31 |
|  | *Cunninghamia lanceolata* | Taxodiaceae | 0.28 |
|  | *Quercus fabri* | Fagaceae | 0.19 |
|  | *Ligustrum lucidum* | Oleaceae | 0.19 |
|  | *Myrsine africana* | Myrsinaceae | 1 |
|  | *Viburnum chinshanense* | Caprifoliaceae | 1 |
|  | *Smilax china* | Liliaceae | 0.78 |
|  | *Lindera glauca* | Lauraceae | 0.78 |
|  | *Rosa cymosa* | Rosaceae | 0.78 |

**Table S2** Summary of the first two axes of the PCA for each soil resource availability and heterogeneity in karst shrubland and woodland. S-Avai: soil resource availability in shrubland; S-Hete: soil resource heterogeneity in shrubland; W- Avai: soil resource availability in woodland; W-Hete: soil resource heterogeneity in woodland.

| Habitat | Environment  variables | Eigenvalue | | | Cumulative proportion | |
| --- | --- | --- | --- | --- | --- | --- |
|  |  | Axis 1 | Axis 2 | Axis 1 | | Axis 2 |
| Shrubland | S- Avai | 2.27 | 1.80 | 36.86 | | 59.91 |
|  | S-Hete | 2.04 | 1.55 | 29.83 | | 47.01 |
| Woodland | W- Avai | 2.78 | 1.49 | 55.18 | | 70.98 |
|  | W-Hete | 2.06 | 1.60 | 30.40 | | 48.64 |

**Table S3** Direct, indirect and total standardized effects of each soil resource availability on abundance and species diversity of shrubland presented in Fig.6a and Fig.6b, respectively. For abbreviations, see Material and Methods and Table 2.

| SEM model | Explanatory variable | Paths to shrubland community | Effect |
| --- | --- | --- | --- |
| Model in Fig.6a  Abundance | SD | Indirect effect | -0.14 ns |
|  |  | Total effect | -0.14 ns |
|  | RBR | Direct effect | 0.34^*^ |
|  |  | Total effect | 0.34^*^ |
|  | TC | Direct effect | 0.15 ns |
|  |  | Total effect | 0.15 ns |
|  | TN | Direct effect | 0.67^**^ |
|  |  | Total effect | 0.67^**^ |
|  | W | Indirect effect | 0.67^**^ |
|  |  | Total effect | 0.67^**^ |
|  | ρb | Direct effect | 0.13 ns |
|  |  | Total effect | 0.13 ns |
|  | | | |
| Model in Fig.6b | SD | Direct effect | 0.41^*^ |
| Species diversity |  | Indirect effect | -0.18^*^ |
|  |  | Total effect | 0.23^*^ |
|  | pH | Direct effect | 0.43 ns |
|  |  | Indirect effect | -0.08 ns |
|  |  | Total effect | 0.35 ns |
|  | TN | Direct effect | 0.77^***^ |
|  |  | Total effect | 0.77^***^ |
|  | W | Indirect effect | 0.72^***^ |
|  |  | Total effect | 0.72^***^ |
|  | ρb | Direct effect | -0.08 ns |
|  |  | Total effect | -0.08 ns |

**Table S4** Direct, indirect and total standardized effects of each soil resource availability on abundance and species diversity of shrub and tree layers of woodland presented in Fig.6c, Fig.6d, Fig.6e and Fig.6f, respectively. For abbreviations, see Material and Methods and Table 2.

| SEM model | Explanatory variable | Paths to woodland community | Effect |
| --- | --- | --- | --- |
| Model in Fig.6c  For shrub layer  Abundance | SD | Indirect effect | 0.37^***^ |
|  |  | Total effect | 0.37^***^ |
|  | pH | Indirect effect | -0.24^*^ |
|  |  | Total effect | -0.24^*^ |
|  | TN | Direct effect | -0.48^***^ |
|  |  | Total effect | -0.48^***^ |
|  | TK | Direct effect | -0.36^**^ |
|  |  | Total effect | -0.36^**^ |
|  | | | |
| Model in Fig.6d | SD | Indirect effect | -0.18^*^ |
| For shrub layer |  | Total effect | -0.18^*^ |
| Species diversity | pH | Indirect effect | 0.21^**^ |
|  |  | Total effect | 0.21^**^ |
|  | TN | Direct effect | 0.08 ns |
|  |  | Total effect | 0.08 ns |
|  | TK | Direct effect | 0.41^**^ |
|  |  | Total effect | 0.41^**^ |
|  | ρb | Direct effect | -0.34^*^ |
|  |  | Total effect | -0.34^*^ |
|  | | | |
| Model in Fig.6e | SD | Direct effect | -0.43 ns |
| For tree layer |  | Indirect effect | 0.41^***^ |
| Abundance |  | Total effect | 0.41^***^ |
|  | pH | Direct effect | -0.61^***^ |
|  |  | Total effect | -0.61^***^ |
|  | TN | Direct effect | 0.08 ns |
|  |  | Total effect | 0.08 ns |
|  | Mg | Indirect effect | -0.51^***^ |
|  |  | Total effect | -0.51^***^ |
|  | ρb | Direct effect | 0.73^***^ |
|  |  | Total effect | 0.73^***^ |
|  | | | |
| Model in Fig.6f | SD | Direct effect | -0.62 ns |
| For tree layer |  | Indirect effect | 0.61^*^ |
| Species diversity |  | Total effect | 0.61^*^ |
|  | pH | Direct effect | 0.25 ns |
|  |  | Indirect effect | -0.42^*^ |
|  |  | Total effect | -0.42^*^ |
|  | TN | Direct effect | -0.80^*^ |
|  |  | Total effect | -0.80^*^ |
|  | TK | Direct effect | -0.49^**^ |
|  |  | Total effect | -0.49^**^ |
|  | ρb | Direct effect | 0.25 ns |
|  |  | Total effect | 0.25 ns |
